# Collaborative mental health care: A narrative review

**DOI:** 10.1097/MD.0000000000032554

**Published:** 2022-12-30

**Authors:** Christopher Reist, Incia Petiwala, Jennifer Latimer, Sarah Borish Raffaelli, Maurice Chiang, Daniel Eisenberg, Scott Campbell

**Affiliations:** a University of California, Irvine, Irvine, CA; b Veterans Affairs Long Beach Healthcare System, Long Beach, CA; c Carbon Health Technologies, Inc, Oakland, CA; d University of California, Los Angeles, Los Angeles, CA; e Department of Psychiatry & Behavioral Neurosciences, Cedars-Sinai Medical Care Foundation, Los Angeles, CA.

**Keywords:** care management, CoCM, collaborative care, integrated care models, measurement based care, mental health, primary care, psychiatry, screening

## Abstract

The Collaborative Care model is a systematic strategy for treating behavioral health conditions in primary care through the integration of care managers and psychiatric consultants. Several randomized controlled trials have demonstrated that Collaborative Care increases access to mental health care and is more effective and cost efficient than the current standard of care for treating common mental illnesses. Large healthcare systems and organizations have begun to adopt Collaborative Care initiatives and are seeing improved treatment outcomes and provider and patient satisfaction. This review discusses current research on the effectiveness and cost-efficiency of Collaborative Care. In addition, this paper discusses its ability to adapt to specific patient populations, such as geriatrics, students, substance use, and women with perinatal depression, as well as the significance of measurement-based care and mental health screening in achieving improved clinical outcomes. Current data suggests that Collaborative Care may significantly improve patient outcomes and time-to-treatment in all reviewed settings, and successfully adapts to special patient populations. Despite the high upfront implementation burden of launching a Collaborative Care model program, these costs are generally offset by long term healthcare savings.

## 1. Introduction

Among the various models of integrated mental health care, the Collaborative Care model (CoCM) stands out as an evidence-based way to improve patient outcomes, team collaboration, and provider satisfaction in primary care settings, with more than 80 randomized controlled trials supporting its efficacy across multiple psychiatric conditions.^[1]^ CoCM can play a crucial role in increasing access to mental health care within the primary care setting, where only 50% of patients with a mental health disorder are recognized, and only 12.5% of those are properly treated.^[2]^ Patients treated with collaborative interventions reach a diagnosis and initiate treatment within 6 months 75% of the time; this is in contrast to treatment as usual, where less than 25% of patients receive appropriate care within the same time frame.^[3]^ Importantly, a recent review of randomized controlled trials examining remote CoCM teams found 9 published studies that collectively support the effectiveness of the model in treating a range of behavioral health conditions, including many mood and anxiety disorders.^[4]^ The importance of integrated mental health is now more relevant than ever, with President Joe Biden emphasizing the importance fully integrated and accessible behavioral and physical healthcare in his 2022 Strategy to Address our National Mental Health Crisis.^[5]^

CoCM has been shown to improve access to behavioral health services, deliver patient-centered behavioral and physical health care in the same setting, and improve overall clinical outcomes.^[1]^ Critical to achieving these benefits are 5 key components: population-based care, measurement-based care (MBC), care management, psychiatric consultation, and brief evidence-based psychotherapy.^[2]^ While each component is crucial, MBC is of particular importance; by itself, incorporating MBC strategies has been linked to improved patient outcomes and faster treatment times.^[3,4]^

Several models of primary care-based collaborative mental health treatment exist, including: Screening, Brief Intervention and Referral to Treatment, the Collaborative Chronic Care Model, Primary Care Behavioral Health, and Co-location of Services.^[6]^ While having some similarities to these models, the CoCM model is unique in that it focuses resources on an identified patient population suffering from mental health concerns, utilizes an integrated care manager with mental health training, and incorporates decision-support and case review by a psychiatrist. The CoCM model relies on algorithmic, stepped care with systematic follow-up and monitoring of patients.^[7,8]^ Furthermore, CoCM specifically relies on close coordination and communication between medical and mental health providers, whereas other models such as Co-location of Services do not afford the same depth of relationship.^[9]^

**Figure 1.**
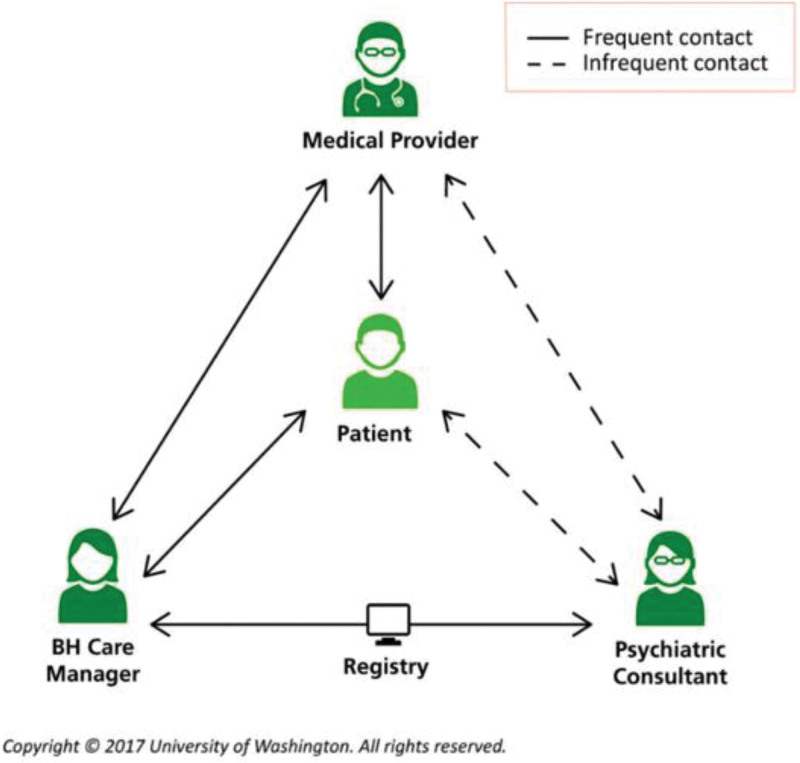
Typical pathway for CoCM. Reprinted with permission from the University of Washington Advanced Integrated Mental Health Solutions Center. CoCM = Collaborative Care model.

In the typical CoCM, a Behavioral Health Care Manager (BHCM) serves as the lynchpin of the program. When the Primary Care Provider (PCP) initiates a patient referral into CoCM, the BHCM performs an initial evaluation to ascertain the presenting problem and create a provisional diagnosis to review with the consulting psychiatrist. The BHCM utilizes a registry to keep track of all patients and prioritize those for regular review. After conducting a case review with the consulting psychiatrist, the BHCM provides the suggested treatment plan to the referring PCP, who reviews and executes upon the treatment plan. (Fig. 1)^[12]^ Concurrently, BHCMs can provide brief, evidence-based psychotherapy, including problem-solving therapy, motivational enhancement therapy, or behavioral activation in addition to brief psychosocial interventions to support treatment; these interventions may include encouraging medication adherence, scheduling follow-ups, and providing referrals to specialty care as appropriate. In addition, the BHCM is tasked with implementing and coordinating other care recommendations, longitudinal symptom monitoring and liaising with both the PCP and consulting psychiatrist. If implemented with these core components, this model of care can improve both mental and physical outcomes, with little to no net change in primary health care costs.^[10]^ In fact, real world studies suggest that CoCM can substantially reduce overall cost of care by improving clinical outcomes. At Blue Cross Blue Shield of Michigan, the average time for participants in their program to reach remission from depression was 16 weeks, compared with 52 weeks for traditional direct care approaches. In addition, Blue Cross Blue Shield of Michigan is tracking towards a 2-3x reduction in medical spending for enrolled patients within 3 years across 190 clinics.^[11]^

CoCM improves the patient experience by allowing for care to be delivered in a “down the hall” manner, with the patient working with known providers already trusted with managing other medical issues. This can address the stigma that some patients may experience and reduce the opportunity for noncompliance with treatment. Much of the existing literature discusses the implementation and outcomes of CoCM in the primary care context. Beyond traditional primary care populations, however, there are specific groups that may benefit from CoCM, including college students, obstetrics and gynecology (OBGYN) patients, geriatric patients, and those in substance abuse treatment programs. Although less studied in these settings, CoCM has been demonstrated to be effective for populations in which mental health needs are inconsistently addressed. In this review, we discuss the unique adaptations of CoCM and the evidence supporting its cost effectiveness in these populations in addition to the important role of MBC and screening.

## 2. Methods

The literature search for this review was performed through a comprehensive overview of multi-disciplinary journal databases and subject specific databases pertaining to Collaborative Care in primary health care settings. Articles used included academic peer reviewed clinical, meta-analysis and observational studies. Other types of content such as government information was briefly used to gather background information. Search terms used to find literature included article keywords, the special populations discussed in this paper, and title words. After a thorough literature search on clinical and economic effects of collaborative care, data was collated to discuss the efficacy of collaborative care on specific populations.

## 3. Discussion

### 3.1. Student health: expanding access and utilization of mental healthcare in university settings

As with primary care, there is a significant unmet need for mental health services in collegiate settings. It is estimated that 17% of students experience serious psychological distress^[13]^ but less than half receive treatment.^[14]^ This is not surprising given that half of adult psychiatric illnesses, including major depression, anxiety disorders, and substance abuse, start by age 14, with 75% presenting by age 25.^[15]^ These individuals face persistent symptoms that negatively impact academic performance, graduation rates, and future income, and lead to increased rates of substance misuse and social dysfunction.^[16]^ Alarmingly, trends show a worsening of mental health among college students in recent years.^[17]^ Two large databases (the National College Health Assessment and the Healthy Minds Study) showed rates of depression, anxiety, nonsuicidal self-injury, suicidal ideation, and suicide attempts markedly increased from 2007 to 2018, with rates doubling over that period in many cases. The steepest increase was observed between 2014 and 2018. This highlights the need to better understand the barriers students face. One factor to consider is how the availability of on campus services affects utilization. A survey of nearly 40,000 students found that of the 20% of students who had used mental health services while attending college, half used on-campus resources, whereas the other half used off-campus services. Older, white, full-time, and female students were more likely to use these services.

As a model to address some of these challenges, CoCM is becoming increasingly widespread in collegiate settings. A survey conducted in 2007 found that 26% of respondents identified their institution as actively implementing some form of integrated behavioral healthcare system on campus. A more recent study including mostly 4-year public and private schools^[18]^ reported that this has now risen to 46%. Many nonintegrated student health centers have reported making referrals to specialty mental health care and coordinating care between departments despite not having formal Collaborative Care services. However, there were still lower levels of shared treatment planning, clinical collaboration, and information sharing being reported in nonintegrated centers as compared to explicitly integrated centers.

Universities and colleges nationwide have increasingly begun to interrogate the benefits of Collaborative Care. One example is the National College Depression Partnership at New York University, which demonstrated that the Collaborative Chronic Care Model for depression can be implemented successfully in campus health centers through a learning collaborative approach.^[19]^ The initiative involved a cumulative total of over 40 colleges and universities starting in 2006 but has not been active in recent years.

While there are no studies that examine the cost effectiveness of CoCM programs specifically in student health settings, providing mental health treatment can yield economic benefits in the form of increased graduation rates. One report estimated that for every dollar invested in prevention and early intervention programs, there was a net societal benefit of $6.49.^[20]^ The Healthy Minds initiative has developed a return-on-investment tool to assist colleges in understanding the benefits of implementing comprehensive mental health services, including CoCM.^[21]^

In summary, there is a tremendous and growing need for mental health services in the college setting. A report by the American Council on Education^[22]^ made 4 recommendations for colleges and universities regarding mental health on campus. Three of these are explicitly addressed by CoCM: implementing routine assessment, enhancing accessibility of clinical services, and integrating mental health promotion and prevention. Additionally, a recent Consensus Report by the National Academies, Sciences, Engineering, and Medicine on student mental health included the following recommendation: “colleges and universities should make behaviorally focused mental health services more readily available in primary care settings to facilitate students’ access to care and improve coordination between mental health and primary care providers, both on campus and in telehealth services.”

### 3.2. Women’s health: perinatal depression as a case study in the application of CoCM

During the childbearing years, many women receive primary care services through an OBGYN provider. These services include routine monitoring of labs, health screening and maintenance of immunizations. To this end, women’s health settings present ample opportunities for CoCM, given the parallels to primary care. Perinatal depression (PPD), however, is unique to women’s health and is a common but underdiagnosed complication of childbirth that affects as many as 23% of women. There has been renewed focus on PPD because of its high prevalence and due to the emergence of new treatments. Therefore, this serves as a good example for discussing the potential impact of the CoCM. PPD is associated with pregnancy complications, impaired maternal-infant bonding, and a host of other negative consequences for both mother and child. The suicide rate has been estimated to be between 2.0 and 3.7 deaths per 100,000 live births^[23]^ and is a leading cause of maternal mortality in the first 12 months postpartum. PPD often goes unrecognized because changes in sleep, appetite and libido can be attributed to normal pregnancy and postpartum changes. Even when PPD is identified, patients receive insufficient or no treatment. In a study of 122 women in a cohort of 1125 postpartum women who were diagnosed with PPD, only 12% had received psychotherapy and 3% had received medication at the 3 month follow-up following initial diagnosis.^[24]^ While most obstetricians recognize the value of managing depression, they lack the tools required for screening and the training and resources needed for follow up care, often relying on specialty referrals, which oftentimes lack availability.^[25]^ Most women between the ages of 18 and 44 have limited access to specialized care; the majority only have access to OBGYNs or PCPs, making CoCM an attractive integrated treatment solution.^[26]^

CoCM has been successfully implemented in several women’s health settings. The MOMCare intervention is a Collaborative Care strategy that has received the most research attention among this population. This approach was tested in the Medicaid population and differs from standard interventions that are typically provided by a team of public health social workers, nurses and nutritionists, who oftentimes inconsistently screen mothers for depressive symptoms.^[27]^ Instead, the MOMCare intervention functions similarly to the traditional CoCM model, with care managers closely collaborating with the patient and a psychiatrist on medication and therapy management, while also monitoring patient progress throughout the maternal journey.^[27]^ When compared to standard treatment, MOMCare is more effective in guiding patients to remission, reducing the severity of depression, and enhancing patient satisfaction; 48% of patients receiving MOMCare achieved or sustained remission for PPD.^[27]^ In addition, providers expressed satisfaction seeing their patients follow through with their referrals and receive more specialized care.^[28]^ Similar findings have been reported from studies using other collaborative approaches, with women reporting less depression symptoms, higher treatment satisfaction, and more successful antidepressant therapy. This is likely because, despite their comfort in screening for PPD, OBGYNs assess their confidence in treating depression and providing antidepressant advice as less than internists or family doctors.^[25]^

Although several studies demonstrate CoCM’s superior clinical outcomes for PPD as compared to treatment as usual, CoCM is oftentimes more expensive, with MOMCare costing approximately $1737 per course of treatment, while the direct cost of care without specialized intervention is approximately $570.^[26]^ Patient monitoring and care management are significant components of CoCM, hence, the increased costs associated with staffing.^[25]^ Despite the higher cost, women who received MOMCare experienced more depression-free days than their counterparts.^[26]^ While the value of a depression-free day can vary across groups, a study by Epperson^[29]^ showed that mothers with untreated PPD had significantly higher annual direct total all-cause medical and pharmaceutical spending than matched controls without PPD ($19,611 vs $15,410), driven primarily by more outpatient visits. When examined more broadly, PPD had an impact on the entire household, not just on the affected mother. This translates into significant all-cause family medical and pharmaceutical spending during the first year following childbirth ($36,049 vs $29,448) and an average of 16 more outpatient visits across the family unit as compared with unaffected households.

In summary, an integrated approach to the care for women with perinatal depression has substantial benefits. Evidence indicates improved clinical outcomes, patient and provider satisfaction, and overall quality of care. Although a collaborative approach may have higher direct costs than the current standard of care, this expense is minimal compared to the impact PPD has on the total cost of care, and the long term burden that untreated PPD has on a mother and her family.

### 3.3. Geriatric health: CoCM as a key part of a whole health solution

The primary care settings are often ideal for screening and treating mental health conditions among older adults. Amongst the geriatric population, nearly 30% report depression or anxiety to their PCPs, with the greatest burden in Hispanic and Asian populations (32–35). Many older adults have a stigmatized perception of mental health treatment and are therefore reluctant to seek specialized care.^[30]^ As a result, primary care presents a critical opportunity for the detection and treatment of mental health disorders in older adults. Like OBGYNs, providers in geriatrics often lack training and time to diagnose and treat mental health conditions, creating another barrier for patients needing care. Studies have shown that CoCM can effectively address this care gap.^[31–35]^

In the last decade, CoCM has been increasingly utilized to care for older adults within primary care. A 2006^[36]^ study demonstrated that older participants assigned to a collaborative intervention reported a 23% reduction in their depressive symptoms, better adherence to medication, and improved satisfaction and quality of life compared to those receiving standard care. The Bridging Resources of an Interdisciplinary Geriatric Health Team via Electronic Networking (BRIGHTEN) program is another well-known study that supports the efficacy of CoCM. This program integrated empirically supported primary care collaboration approaches and tailored them for delivery to a geriatric population via in-person and/or virtual care. The BRIGHTEN intervention utilized a virtual interdisciplinary team to screen and treat depression in older adults in outpatient primary and specialty medical clinics. Key findings included an increased number of self-referring patients, a significant decrease in depressive symptoms, and improved communication among providers.^[34]^

The BRIGHTEN initiative has also demonstrated improved minority participation in mental health treatment, which has historically proven to be challenging. nonwhite elders, the majority of geriatric patients, are more prone to reporting somatic complaints, and hold negative views on mental health diagnoses and treatment. Instead of seeing a specialist, these adults felt more comfortable entering a program through primary care.^[31,34]^ Importantly, older people who received Collaborative Care reported feeling listened to and cared for, and some rated these positive interactions as more effective than pharmacotherapy.^[32]^

Beyond improved clinical outcomes, there is evidence that using CoCM when treating elderly patients with neurocognitive disorders, which frequently coexist with behavioral disorders, can reduce medical costs and save provider time.^[37]^ Early identification of mental health conditions can lower costs for health systems and patients in the form of reduced hospital admissions, emergency visits, and more drastic medical interventions later in the course of the disease.^[38]^ In addition, CoCM has been associated with lower ambulatory cost in elderly populations.^[39]^ While implementation of CoCM can increase overall healthcare costs in the first year, that figure drops in later years, suggesting that an early investment in mental health care results in long term cost savings.^[40]^

In summary, Collaborative Care appears to be an effective intervention to improve clinical outcomes in any population. CoCM also offers greater mental health accessibility and opportunities for better detection of mental health conditions. Additional research is required to assess patient outcomes and provider experience using Collaborative Care in a geriatric setting to provide a holistic account of the benefits and challenges.

### 3.4. Treatment for substance abuse

Nearly 20 million people in the United States suffer from a substance abuse disorder and do not receive adequate treatment. Those who seek treatment mostly do so in the primary care setting.^[41]^ This presents an opportunity for the millions of patients who do not otherwise receive treatment due to inadequate access to care and stigma. Patients receiving substance abuse treatment commonly have comorbid psychiatric conditions such as depression and anxiety. Treatment of these conditions has been shown to improve health outcomes related to substance abuse.^[42]^ Early diagnosis and behavioral intervention can result in faster treatment times and higher remission rates. Numerous studies have suggested that integrated treatment for comorbid mental health conditions is superior to individual, siloed treatment plans.^[43]^

A team-based approach that involves a BHCM, consulting psychiatrist, PCP, and substance use care manager is necessary for CoCM to be effective.^[44]^ In some cases, the behavioral and substance use care manager can be the same person if they are trained appropriately. CoCM functions well in this setting because PCPs tend to lack the time, resources and skill with behavioral interventions required for long term treatment with a substance abuse patient.^[44]^ PCPs frequently refer their patients to substance abuse specialists. However, fewer than 35% of patients follow through with these referrals, with patients citing clinical differences in treatment decisions and varying wait times as reasons they do not follow through.^[45]^ Thus, there is a significant opportunity to increase access to care and compliance using CoCM.

One of the unique advantages of CoCM is that it targets substance abuse and behavioral health symptoms simultaneously through 2 collaborative providers on the same team, reducing the burden on the PCP, and improving collaboration.^[44]^ It is no surprise, therefore, that 86% of primary care practices agree that training related to substance abuse treatment would be helpful for their clinical staff.^[41]^ CoCM for substance abuse can increase the number of patients treated by giving PCPs more time and resources^[44]^; in fact, 1 study reported a 375% increase in patients treated in primary care once CoCM was implemented.^[46]^

In addition, patients treated for substance abuse via CoCM report greater abstinence from alcohol and drugs than patients receiving treatment as usual.^[47]^ Individuals participating in a trial on opioid addiction treated with Buprenorphine and a collaborative intervention were more likely to have successful outcomes and remain in treatment as compared to patients treated with Buprenorphine alone.^[48]^ One caveat, however, is that as a result of increased staffing needs, a health system must be prepared for a near-term increase in their costs for the employment and training of care managers. While there is currently limited research examining the cost effectiveness of CoCM in treating substance abuse, studies demonstrating higher patient remission rates suggest that CoCM lowers the total cost of care in the long run.

In summary, research has demonstrated that CoCM is effective in treating substance abuse in primary care settings. Patients reported increased access to care and clinicians noted higher remission rates. More research is required to assess CoCM’s cost effectiveness for patients with substance abuse problems.

### 3.5. Measurement-based care and screening

Many people diagnosed with mental health conditions never receive treatment, which is a common finding across all the populations examined in this paper. Although CoCM has been shown to improve access to care, we suggest that the first step in addressing the treatment gaps is MBC and population-level screening. Numerous studies have demonstrated that routine screening for mental disorders enables early detection and intervention. Current efforts to improve screening include the utilization of different screening tools such as the Patient Health Questionnaire (PHQ-2 and PHQ-9).^[49]^ A meta-analysis of 14,760 adults has validated the use of the PHQ-2 and PHQ-9 as reliable and effective measures to detect depression in primary care.^[50]^

Equally important to screening, MBC can be described as the evaluation of patient symptoms at any stage of treatment to inform treatment choices.^[51]^ MBC acts as a critical component of any population health strategy and is an accepted practice for many medical conditions such as diabetes or hypertension, where objective measures reflecting the state of health of the population are easily available. Challenges to the routine use of MBC include the complex processes required to initiate systematic routine data collection and administrative burden such as increased time and paperwork combined with limited resources.^[52]^ In addition, barriers to implementing MBC have been identified at both the patient (e.g., concerns about how information is being used) and provider level (e.g., over-valuing clinical judgment).

Ultimately, both these components are essential to gauge CoCM’s effectiveness as they enable clinicians and care managers to continuously monitor patient progress. Monitoring patient data consistently leads to better results, increased patient-provider contact, and increased treatment fidelity. For instance, reliance on clinical judgment may fall short in identifying poor treatment responses, which could result in the continuation of ineffective treatment. However, if screening and assessment of patient symptoms are performed frequently and consistently, the treatment plans can be adjusted when necessary.

## 4. Conclusion

Launching CoCM requires investment in population health tools such as registry and tracking systems, low-burden population screening software, and interoperability with legacy electronic medical record systems. In addition, adequate staffing is critical; providers must be trained on CoCM, BHCMs hired, and consulting psychiatrists identified, suggesting a considerable investment of time and money. However, these upfront costs are generally offset by longer-term health care savings vis-a-vis lower overall healthcare utilization, better medical treatment adherence, etc. As CoCM becomes more widely accepted and implemented, reimbursement is becoming more universal, with CMS and most commercial payers approving reimbursement for CoCM services. Organizations can use this growing emphasis on collaborative interventions to implement these novel, evidence-based, and cost-effective models of care. CoCM presents opportunities for healthcare systems to have a greater impact on clinical outcomes and therefore a greater ability to take on risk. It is critical that payors consider these services preventative, reducing the cost burden and barriers to entry for enrollment. Early data suggests that enrollment and treatment compliance increase by over 50% by eliminating patient financial responsibility (such as copayment or coinsurance).

The unique strength of CoCM is its ability to adapt to the unique concerns of specific populations, such as students, geriatric patients, women’s health, and substance abuse treatment. Historically, it has been difficult to diagnose and treat mental health conditions in these populations. Fortunately, CoCM provides innovative treatment options that enhance patient access and outcomes. In addition, the use of MBC and screening contribute to the effectiveness of CoCM by promoting early intervention and ongoing treatment.

In the pursuit of the Quadruple Aim of Healthcare, CoCM has the potential to play a significant role and continues to gain momentum as an evidence-based model of care. At its core, CoCM is a population health strategy that delivers value-based care with a focus on patient experience. Studies of CoCM have shown increased provider satisfaction and increased provider confidence in managing behavioral health problems. The implementation of CoCM lags far behind given the substantial body of empirical evidence supporting its use. Greater implementation of CoCM in a variety of clinical settings may be a creative solution to the growing mental health pandemic.

## Author contributions

**Conceptualization:** Christopher Reist, Maurice Chiang.

**Project administration:** Christopher Reist, Maurice Chiang, Incia Petiwala.

**Supervision:** Christopher Reist, Maurice Chiang, Daniel Eisenberg, Scott Campbell.

**Writing – original draft:** Christopher Reist, Incia Petiwala, Maurice Chiang.

**Writing – review & editing:** Christopher Reist, Incia Petiwala, Jennifer Latimer, Sarah Borish, Maurice Chiang, Daniel Eisenberg, Scott Campbell.

## References

[1] Huffman JC Niazi SK Rundell JR. Essential articles on collaborative care models for the treatment of psychiatric disorders in medical settings: a publication by the *Academy of Psychosomatic Medicine Research and Evidence-Based Practice Committee*. Psychosomatics. 2014;55:109–22. 24370112 10.1016/j.psym.2013.09.002

[2] Kroenke K Unutzer J. Closing the false divide: sustainable approaches to integrating mental health services into primary care. J Gen Intern Med. 2017;32:404–10. 28243873 10.1007/s11606-016-3967-9 PMC5377893

[3] Tong G Xiang Y-T Xiao L. Measurement-based care versus standard care for major depression: a randomized controlled trial with blind raters. Am J Psychiatry. 2015;172:1004–13. 26315978 10.1176/appi.ajp.2015.14050652

[4] Leonard B Kelley SD Breda Regina de Andrade CA. Riemer is with the Department of Psychology, Wilfrid Laurier University, Waterloo, Ontario, Canada. Effects of routine feedback to clinicians on mental health outcomes of youths: results of a randomized trial. Psychiatr Serv. 2011;62:1423–9. 22193788 10.1176/appi.ps.002052011

[5] Bagalman E Dey J Jacobus-Kantor L. HHS roadmap for behavioral health integration. ASPE. 2022;1:1–9.

[6] Babor TF Del Boca F Bray JW. Screening, brief intervention and referral to treatment: implications of SAMHSA’s SBIRT initiative for substance abuse policy and practice. Addiction. 2017;112:110–7. 28074569 10.1111/add.13675

[7] Bodenheimer T Wagner EH Grumbach K. Improving primary care for patients with chronic illness the chronic care model, part 2. JAMA. 2002;288:1909–14. 12377092 10.1001/jama.288.15.1909

[8] Eisenberg D Hunt J Speer N. Help seeking for mental health on college campuses: review of evidence and next steps for research and practice. Harv Rev Psychiatry. 2012;20:222–32. 22894731 10.3109/10673229.2012.712839

[9] Anderson K Balderrama SR Davidson J. Considerations for integration of counseling and health services on College and University Campuses. J Am Coll Health. 2010;58:583–96. 20452936 10.1080/07448481.2010.482436

[10] Kilbourne AM Prenovost KM Liebrecht C. Randomized controlled trial of a collaborative care intervention for mood disorders by a National Commercial Health Plan. Psychiatry Serv. 2019;70:219–24. 10.1176/appi.ps.201800336 PMC6522242 30602344

[11] Richmond L. Payors train PCPs to treat mental health in house. Available at: https://psychnews.psychiatryonline.org/doi/10.1176/appi.pn.2021.11.4 [access date July 19, 2022].

[12] University of Washington. Team structure. Available at: https://aims.uw.edu/collaborative-care/team-structure. [accessed August 26, 2022].

[13] Blanco C Okuda M Wright C. Mental health of college students and their non-college-attending peers: results from the National Epidemiologic Study on Alcohol and Related Conditions. Arch Gen Psychiatry. 2008;65:1429–37. 19047530 10.1001/archpsyc.65.12.1429 PMC2734947

[14] Eisenberg D Hunt J Speer N. Mental health service utilization among college students in the United States. J Nerv Ment Dis. 2011;199:301–8. 21543948 10.1097/NMD.0b013e3182175123

[15] Kessler RC Berglund P Demler O. Lifetime prevalence and age-of-onset distributions of DSM-IV disorders in the national comorbidity survey replication. Arch Gen Psychiatry. 2005;62:593–602. 15939837 10.1001/archpsyc.62.6.593

[16] Lisa S-P Woodbridge MW Mendelsohn J. Factors affecting mental health service utilization among California Public College and University Students. Psychiatr Serv. 2016;67:890–7. 27032662 10.1176/appi.ps.201500307

[17] Duffy ME Twenge JM Joiner TE. Trends in mood and anxiety symptoms and suicide-related outcomes among U.S. Undergraduates, 2007–2018: evidence from two national surveys. J Adolesc Health. 2019;65:590–8. 31279724 10.1016/j.jadohealth.2019.04.033

[18] Readdean KC Heuer AJ Hoban MT. Integrated primary care behavioral health services in college health: results from a national survey of health center administrators. J Am Coll Health. 2021;69:478–87. 31702958 10.1080/07448481.2019.1681432

[19] Chung H Klein MC Silverman D. A pilot for improving depression care on college campuses: results of the college breakthrough series–depression (CBS-D) project. J Am Coll Health. 2011;59:628–39. 21823958 10.1080/07448481.2010.528097

[20] Ashwood JS Stein BD Briscombe B. Payoffs for California College students and taxpayers from investing in student mental health. Rand Health Q. 2016;5:11. 10.7249/rhq-05-04-11 PMC5158226 28083421

[21] Return on Investment Calculator (R.O.I.) for College Mental Health Services and Programs. Available at: https://umich.qualtrics.com/jfe/form/SV_6xN9QUSlFtgtRQh [access date July 19, 2022].

[22] Lipson SK Abelson S Ceglarek P. Investing in student mental health: opportunities & benefits for college leadership. Am Coun Educ. 2019;1:3–10.

[23] Meurk C Wittenhagen L Lucke J. Suicidal behaviours in the peripartum period: a systematic scoping review of data linkage studies. Arch Women’s Mental Health. 2021;24:579–93. 10.1007/s00737-021-01102-x 33742281

[24] Horowitz JA Cousins A. Postpartum depression treatment rates for at-risk women. Nurs Res. 2006;55:S23–7. 16601631 10.1097/00006199-200603001-00005

[25] Melville JL Reed SD Russo J. Improving care for depression in obstetrics and gynecology: a randomized controlled trial. Obstet Gynecol. 2014;123:1237–46. 24807320 10.1097/AOG.0000000000000231 PMC4052378

[26] Grote NK Simon GE Russo J. Incremental benefit-cost of MOMCare: collaborative care for perinatal depression among economically disadvantaged women. Psychiatr Serv. 2017;68:1164–71. 28669288 10.1176/appi.ps.201600411

[27] Grote NK Katon WJ Russo JE. Collaborative care for perinatal depression in socioeconomically disadvantaged women: a randomized trial. Depress Anxiety. 2015;32:821–34. 26345179 10.1002/da.22405 PMC4630126

[28] Grote NK Katon WJ Lohr MJ. Culturally relevant treatment services for perinatal depression in socio-economically disadvantaged women: the design of the MOMCare study. Contemp Clin Trials. 2014;39:34–49. 25016216 10.1016/j.cct.2014.07.001 PMC4226398

[29] Epperson CN Huang M Cook K. Healthcare resource utilization and costs associated with postpartum depression among commercially insured households. Curr Med Res Opin. 2020;36:1707–16. 32696705 10.1080/03007995.2020.1799772

[30] Joo JH Rostov P Feeser S. Engaging an Asian immigrant older adult in depression care: collaborative care, patient-provider communication and ethnic identity. Am J Geriatr Psychiatry. 2021;29:1267–73. 34419363 10.1016/j.jagp.2021.07.009 PMC8808368

[31] Bartels SJ. Why collaborative care matters for older adults in China. Lancet Psychiatr. 2015;2:286–7. 10.1016/S2215-0366(15)00049-8 26360064

[32] Li LW Xue J Conwell Y. Implementing collaborative care for older people with comorbid hypertension and depression in rural China. Int Psychogeriatr. 2020;32:1457–65. 31630703 10.1017/S1041610219001509 PMC7170762

[33] Ng TP Nyunt MSZ Feng L. Collaborative care for primary care treatment of late-life depression in Singapore: randomized controlled trial. Int J Geriatr Psychiatry. 2020;35:1171–80. 32453449 10.1002/gps.5353

[34] Emery EE Lapidos S Eisenstein AR. The BRIGHTEN program: implementation and evaluation of a program to bridge resources of an interdisciplinary geriatric health team via electronic networking. Gerontologist. 2012;52:857–65. 22437330 10.1093/geront/gns034

[35] Emery-Tiburcio E Mack L Lattie EG. Managing depression among diverse older adults in primary care: the BRIGHTEN program. Clin Gerontol. 2017;40:88–96. 28452672 10.1080/07317115.2016.1224785

[36] Hunkeler EM Katon W Tang L. Long term outcomes from the IMPACT randomised trial for depressed elderly patients in primary care. BMJ. 2006;332:259–63. 16428253 10.1136/bmj.38683.710255.BE PMC1360390

[37] Lim CT Rosenfeld LC Nissen NJ. Remote care management for older adult populations with elevated prevalence of depression or anxiety and comorbid chronic medical illness: a systematic review. J Acad Consult Liaison Psychiatr. 2022;63:198–212. 10.1016/j.jaclp.2022.02.005 35189427

[38] Heintz H Monette P Epstein-Lubow G. Emerging collaborative care models for dementia care in the primary care setting: a narrative review. Am J Geriatr Psychiatry. 2020;28:320–30. 31466897 10.1016/j.jagp.2019.07.015

[39] Katon WJ Schoenbaum M Fan M. Cost-effectiveness of improving primary care treatment of late-life depression. Arch Gen Psychiatry. 2005;62:1313–20. 16330719 10.1001/archpsyc.62.12.1313

[40] Unutzer J Katon WJ Fan MY. Long-term cost effects of collaborative care for late-life depression. Am J Manag Care. 2008;14:95–100. 18269305 PMC3810022

[41] Urada D Teruya C Gelberg L. Integration of substance use disorder services with primary care: health center surveys and qualitative interviews. Subst Abuse Treat Prev Policy. 2014;9:15. 24679108 10.1186/1747-597X-9-15 PMC3978198

[42] Brackett CD Duncan M Wagner JF. Multidisciplinary treatment of opioid use disorder in primary care using the collaborative care model. Subst Abus. 2022;43:240–4. 34086531 10.1080/08897077.2021.1932698

[43] Kelly TM Daley DC. Integrated treatment of substance use and psychiatric disorders. Soc Work Public Health. 2013;28:388–406. 23731427 10.1080/19371918.2013.774673 PMC3753025

[44] Campbell CI Saxon AJ Boudreau DM. PRimary care opioid use disorders treatment (PROUD) trial protocol: a pragmatic, cluster-randomized implementation trial in primary care for opioid use disorder treatment. Addict Sci Clin Pract. 2021;16:9. 33517894 10.1186/s13722-021-00218-w PMC7849121

[45] Patel MP Schettini P O’Leary CP. Closing the referral loop: an analysis of primary care referrals to specialists in a large health system. J Gen Intern Med. 2018;33:715–21. 29532299 10.1007/s11606-018-4392-z PMC5910374

[46] LaBelle CT Han SC Bergeron A. Office-based opioid treatment with Buprenorphine (OBOT-B): statewide implementation of the Massachusetts Collaborative Care Model in Community Health Centers. J Subst Abuse Treat. 2016;60:6–13. 26233698 10.1016/j.jsat.2015.06.010 PMC4682362

[47] Watkins KE Ober AJ Lamp K. Collaborative care for opioid and alcohol use disorders in primary care: the SUMMIT randomized clinical trial. JAMA Intern Med. 2017;177:1480–8. 28846769 10.1001/jamainternmed.2017.3947 PMC5710213

[48] Alford DP LaBelle CT Kretsch N. Collaborative care of opioid-addicted patients in primary care using buprenorphine: five-year experience. Arch Intern Med. 2011;171:425–31. 21403039 10.1001/archinternmed.2010.541 PMC3059544

[49] Siniscalchi KA Broome ME Fish J. Depression screening and measurement-based care in primary care. J Prim Care Commun Health. 2020;11:2150132720931261. 10.1177/2150132720931261 PMC7673056 33185122

[50] Smithson S Pignone MP. Screening adults for depression in primary care. Med Clin North Am. 2017;101:807–21. 28577628 10.1016/j.mcna.2017.03.010

[51] Lewis CC Boyd M Puspitasari A. Implementing measurement-based care in behavioral health: a review. JAMA Psychiatr. 2019;76:324–35. 10.1001/jamapsychiatry.2018.3329 PMC6584602 30566197

[52] Hatfield DR Ogles BM. Why some clinicians use outcome measures and others do not. Admin Pol Mental Health Mental Health Serv Res. 2007;34:283–91. 10.1007/s10488-006-0110-y 17211715

